# Dysregulation of the GPX4/ACSL4 axis mediates ferroptosis in neurons of T2DD rats

**DOI:** 10.3389/fphar.2026.1891812

**Published:** 2026-08-31

**Authors:** Yi Wang, Shuo Wang, Shuai Zhao, Yanli Lei, Yucheng Fan, Xinyi Zheng, Lan Yang, Yanmei Ma

**Affiliations:** 1 School of Basic Medical Sciences, Ningxia Medical University, Yinchuan, China; 2 Department of Pathology, Shenzhen Traditional Chinese Medicine Hospital, The Fourth Clinical Medical College of Guangzhou University of Chinese Medicine, Shenzhen, China; 3 Department of Pathology, School of Basic Medical Science, Guangdong Medical University, Shenzhen, China; 4 Department of Gastrointestinal Surgery, Ningxia Uyghur Autonomous Region People's Hospital, Yinchuan, China; 5 Department of Pathology, Affiliated Shizuishan First People's Hospital of Ningxia Medical University, Shizuishan, China

**Keywords:** diabetes comorbid with depression(T2DD), ferroptosis, GPX4-ACSL4 axis, lipid peroxidation, neuronal injury

## Abstract

**Introduction:**

Diabetes mellitus complicated with depression (T2DD) is different from a single disease. It leads to more severe damage to nerve cells and cognitive dysfunction, and has a poor prognosis. Existing evidence indicates that ferroptosis—a form of programmed cell death driven by iron accumulation and lipid peroxidation—is involved in both diabetes and depression. Glutathione peroxidase 4 (GPX4) and long-chain acyl-CoA synthetase family member 4 (ACSL4) are key regulators of ferroptosis, but their roles in T2DD-associated neural damage remain unclear.

**Methods:**

In this study, we established a T2DD rat model using a high-fat diet combined with streptozotocin injection and chronic unpredictable mild stress and performed behavioral tests to assess depressive-like behaviors. Meanwhile, HT22 hippocampal neuronal cells were exposed to high glucose and corticosterone to simulate an *in vitro* model of T2DD. We investigated the role of the GPX4/ACSL4 axis in ferroptosis in the primary somatosensory cortex.

**Results:**

*In vivo*, T2DD rats exhibited reduced spontaneous activity, aggravated neuronal damage, Fe^2+^ accumulation in the cortical region, elevated oxidative stress, upregulation of CSL4/NCOA4 expression, and downregulation of GPX4/SLC7A11 expression, indicating that T2DD exacerbates neural damage. *In vitro*, high glucose (50 mM) and corticosterone (200 μM) synergistically reduced HT22 cell viability, increased reactive oxygen species (ROS) levels, and induced JC-1 and Ca^2+^ alterations suggesting mitochondrial depolarization and aggravated mitochondrial damage, further complementing the above conclusions. Notably, treatment with the ACSL4 inhibitor AS252424 reversed the abnormalities in reactive oxygen species, Fe^2+^, and malondialdehyde levels induced by RSL3 (a GPX4 inhibitor), while restoring glutathione (GSH) levels and Adenosine Triphosphate suggesting that dysregulation of the GPX4/ACSL4 axis may be the central mechanism underlying ferroptosis and neuronal damage in T2DD.

**Conclusion:**

This study reveals the role of ferroptosis in the T2DD model and provides new insights into therapeutic interventions targeting lipid metabolism pathways.

## Introduction

Diabetes comorbid with depression (T2DD) refers to type 2 diabetes mellitus (T2DM) comorbid with depressive symptoms. It is reported that approximately 25% of T2DM patients have severe depressive symptoms (Prigge et al., 2022; Tinajero and Malik, 2021). Depression typically presents with somatic symptoms such as fatigue, pain, and sleep disturbances, as well as emotional symptoms such as persistent low mood and anhedonia (Chireh et al., 2019). Notably, individuals with depression are 1.5 times more likely to develop diabetes than the general population, and both diabetes and depression can cause varying degrees of neuronal damage in different brain regions (Chireh et al., 2019). Existing studies suggest that diabetes may lead to structural abnormalities in the hippocampus, prefrontal cortex, and amygdala (Choi et al., 2020; Song, 2023; Ho et al., 2013), Correspondingly, depression is associated with morphological and functional deficits in these overlapping neuroanatomical regions (Li J. et al., 2024; Zhang et al., 2018). In animal models of depression, behavioral manifestations include reduced time spent in social areas compared to healthy controls (indicating “avoidance” behavior), reduced sucrose preference, and reduced activity. When diabetes and depression coexist, depressive-like behaviors are exacerbated, implying that this comorbidity may amplify neuronal damage in brain regions associated with behavior, significantly increase the risk of cognitive dysfunction, exacerbate metabolic dysregulation, and reduce treatment adherence (Yan et al., 2010). This pathological synergy may lead to a worsened prognosis in T2DD patients (Li S. et al., 2024; Stuart and Baune, 2012). Therefore, investigating the mechanisms of neuronal damage in T2DD is crucial for developing targeted therapies for patients with both conditions.

Ferroptosis represents a programmed cell death modality driven by iron accumulation, distinguished by lipid oxidative damage and dysregulated redox homeostasis (Wei et al., 2023). In contrast to classical apoptotic, necrotic, or autophagic pathways, ferroptosis arises from redox imbalance marked by excessive reactive oxygen species (ROS) accumulation and dysregulated iron metabolism. Ferroptosis plays a critical role in various diseases (Sun et al., 2024). For example, the antidiabetic drug metformin, when combined with anticancer agents, induces ferroptosis in breast cancer cells (Yang et al., 2021), thereby enhancing therapeutic efficacy. In diabetic cardiomyopathy, cardiomyocytes are more prone to ferroptosis (Wang et al., 2025), leading to poor prognosis. Similarly, diabetes comorbid with hypertension or hyperlipidemia increases the susceptibility of central nervous system neurons to ferroptosis, exacerbating neuronal damage (An et al., 2022). However, the relationship between neuronal injury and ferroptosis in diabetes comorbid with depression remains to be fully elucidated. Given the shared pathology of iron dysregulation and oxidative stress in both diabetes and depression, ferroptosis emerges as a compelling candidate mechanism underlying their synergistic neuronal injury.

Ferroptosis involves complex mechanisms, including iron metabolism, the Xc-/GPX4 pathway, lipid metabolism, the P53 pathway, FSP1/AIFM2, ATG5-ATG7-NCOA4, and glutamine metabolism. GPX4, a selenoprotein enzyme, employs the antioxidant glutathione in its reduced state (GSH) as an essential cofactor to catalyze the conversion of lipid hydroperoxides (LOOH) within cellular membranes into biologically inert lipid alcohols (LOH), thereby preserving the structural integrity of membranes (Wang B. et al., 2024). Acyl-CoA synthetase long-chain family member 4 (ACSL4), a critical intermediate in lipid biosynthesis and an essential enzyme in lipid metabolic pathways, is highly abundant in the brain and demonstrates pronounced expression levels in neural tissues (Ma et al., 2024; Tian et al., 2024; Wei et al., 2023), an essential enzyme in lipid metabolic pathways, demonstrates pronounced expression levels in neural tissues (Tian et al., 2024). This process enhances cellular susceptibility to ferroptosis, accelerates its progression, and ultimately leads to membrane rupture and ferroptotic cell death. Inhibition of GPX4 expression amplifies ACSL4 activity, thereby promoting ferroptosis. Recent studies have demonstrated significant downregulation of GPX4 in both diabetes and depression models (Wang et al., 2023; Xu et al., 2024), alongside dysregulated iron metabolism in the brains of patients with these conditions (Li et al., 2025; Wang et al., 2023). These findings suggest that reduced GPX4 expression and iron homeostasis imbalance may serve as key links between diabetes and depression. However, whether T2DD exacerbates neuronal injury by regulating ferroptosis via the GPX4/ACSL4 axis remains unclear.

In this study, we established a T2DM rat model through high-fat diet and intraperitoneal streptozotocin (STZ) injection. A T2DD model was further developed by subjecting these diabetic rats to chronic unpredictable mild stress (CUMS) (Li et al., 2021). For *in vitro* experiments, HT22 cells were treated with high glucose (HG) and corticosterone (CORT) to simulate T2DD. Behavioral differences in rats were observed, and oxidative stress markers (e.g., ROS, MDA, GSH, SOD) and ferrous iron (Fe^2+^) levels were measured to investigate whether ferroptosis contributes to central nervous system (CNS) injury in T2DD. The relationship between the GPX4/ACSL4 signaling axis and ferroptosis was analyzed to test the hypothesis that dysregulation of the GPX4/ACSL4 axis is a central mechanism driving ferroptosis and neuronal injury in T2DD.

## Materials and methods

### Rationale for animal models

The high-sucrose/high-fat (HSHF) diet combined with low-dose STZ injection is a well-established rodent model of type 2 diabetes mellitus, effectively mimicking insulin resistance and progressive β-cell dysfunction (Srinivasan et al., 2005). The CUMS paradigm is a widely validated model for inducing depression-like behaviors in rodents through prolonged, unpredictable mild stressors, which reliably recapitulates behavioral and neuroendocrine features of clinical depression (Reed et al., 2000; Willner, 2017). The combination of these two models (T2DM + CUMS) has been used to study comorbid diabetes and depression, reflecting the interactive effects of metabolic and psychological stress on neural pathways.

### Animals and grouping

Forty male Wistar rats (age: 8 weeks; initial weight range: 200–230 g) were sourced from Vital River Laboratory (Beijing, China). All procedures were conducted in accordance with the ARRIVE guidelines under ethical approval from Ningxia Medical University’s Institutional Animal Care Committee (IACUC-NYLAC-2021-063), strictly implementing the 3R principles. During a 7-day acclimation period, the rodents were housed in specific pathogen-free (SPF) facilities under controlled environmental conditions, including an ambient temperature of 22 °C ± 1 °C, a relative humidity of 60% ± 5%, and standard 12-h light/dark cycles. Following environmental adaptation, subjects were randomly assigned to four experimental cohorts (n = 10/group) through computer-generated randomization:Control group: Received standard chow without interventions. CUMS group: Exposed exclusively to chronic unpredictable mild stress protocolsT2DM group: Administered a HSHF dietary regimen for 28 days followed by intraperitoneal STZ injection (38 mg/kg; Sigma-Aldrich S0130) post-16 h fasting. T2DD group: Combined T2DM induction and CUMS exposure. Diabetic phenotype validation involved caudal blood collection 72 h post-STZ administration. Inclusion criteria required sustained hyperglycemia (>16.7 mmol/L) across three consecutive daily measurements. Control and T2DD cohorts received isovolumetric PBS injections as vehicle controls.

### CUMS protocol

The CUMS paradigm was implemented over an 8-week period for both CUMS and T2DD cohorts, with daily stressors randomized to prevent habituation. Stimuli included social crowding (6 rats per cage for 24 h), damp bedding (500 mL water added per cage for 24 h), cage tilt (45° inclination for 24 h), mild tail pinch (90 s), forced swimming in ice-cold water (4 °C for 5 min), 24-h fasting, and 12-h nocturnal light exposure. To minimize environmental familiarity, stress procedures were conducted in a dedicated facility separate from the housing environment. Control and T2DM groups maintained *ad libitum* access to food and water without hypoglycemic interventions. Rats meeting predefined inclusion criteria (e.g., stable post-stress physiological parameters) proceeded to subsequent behavioral and histopathological analyses.

### Behavioral assessments

#### Open field test (OFT)

The OFT was performed in a dark-painted wooden arena (100 × 100 × 38 cm) partitioned into a 5 × 5 grid configuration. Animals were individually positioned at the central grid under dim illumination (20 lux) and allowed to freely explore the environment for 5 min, with locomotor activity recorded via an overhead video tracking system. Between trials, the arena was thoroughly sanitized using 75% ethanol to neutralize residual odors. Behavioral metrics, including rearing frequency (vertical movements defined as full extension of hindlimbs), were quantified using automated analysis software (EthoVision XT, Noldus).

#### Forced swimming test (FST)

For the FST, rats were placed in a transparent polycarbonate cylinder (40 cm inner diameter × 100 cm height) filled to 30 cm depth with water maintained at 20 °C ± 1 °C. Each session lasted 5 min, during which immobility duration—defined as the absence of active limb movements beyond minimal efforts to remain buoyant—was scored manually by two blinded observers.

### Sucrose preference test (SPT)

For the SPT, rats were individually housed and habituated to the presence of two drinking bottles for 3 days prior to testing. During the habituation period, both bottles contained tap water. On the test day, following 12 h of food and water deprivation, each rat was presented with two pre-weighed bottles containing 200 mL of 1% (w/v) sucrose solution and 200 mL of tap water, respectively. The bottles were placed on the cage lids, and their positions were counterbalanced every 6 h over the 24-h test session to avoid side bias. Fluid intake was determined by weighing each bottle at the end of the session. Sucrose preference was calculated as the ratio of sucrose solution consumption to total fluid intake (sucrose + water) × 100%. All measurements were performed by two independent observers blinded to the experimental conditions.

### Histological staining

#### H&E staining

Tissue sections were baked at 65 °C for 1 h to enhance adhesion, followed by dewaxing in sequential xylene baths (xylene I and II, 15 min each) and rehydration through graded ethanol solutions (100% I and II, 15 s each; 90%, 75%, 60%, and 50%, 15 s each). After rinsing with distilled water, sections were stained with Mayer’s hematoxylin (10 min), Then, the samples were differentiated in 1% acid alcohol (1% HCl in 70% ethanol) for 2 s, followed by bluing achieved by rinsing under running tap water for 5 min. Counterstaining was carried out with eosin Y for 10 min. The stained sections were then dehydrated through a graded ethanol series (70%–100%), cleared in xylene, and mounted using neutral resin for digital histopathological evaluation.

### Nissl staining

Sections underwent identical dewaxing and hydration steps as H&E staining. Slides were immersed in a 0.1% cresyl violet staining solution and maintained at 37 °C for 30 min. Subsequently, residual dye was eliminated through sequential washing steps, followed by dehydration using an ascending ethanol series. After xylene clearing, slides were imaged using a whole-slide scanner for neuronal integrity assessment.

### Cell culture and treatments

HT22 mouse hippocampal neurons (obtained from Haisheng Biotech, China) were grown in DMEM/F12 medium supplemented with 10% fetal bovine serum (Viva Cell), 100 U/mL penicillin, and 100 μg/mL streptomycin, under standard culture conditions (37 °C, 5% CO_2_). At 80% confluency, cells were assigned to six experimental groups:Control: Basal medium; HG: 50 mM D-glucose; CORT: 200 µM corticosterone; HG + CORT: Combined 50 mM glucose and 200 µM corticosterone; HG + CORT + RSL3: Above treatments +0.8 µM RSL3 (GPX4 inhibitor); HG + CORT + RSL3+AS252424: Additional 10 mM AS252424 (ACSL4 inhibitor). Treatments were administered for 48 h.

### CCK-8 proliferation assay

HT22 cells were seeded into 96-well plates at a density of 10,000 cells per well in 100 µL of culture medium and were left to attach for 24 h. After the experimental treatments, 10 µL of Cell Counting Kit-8 solution (Dojindo, CK04) was added to each well. Optical density measurements at 450 nm were acquired after 4 h using a SpectraMax i3x microplate reader (Molecular Devices) pre-warmed to 25 °C. Viability percentages were calculated relative to untreated controls using nonlinear regression analysis (GraphPad Prism v9.5).

### Fe^2+^ quantification

Tissue/cell lysates were processed on ice, centrifuged (12,000 × g, 10 min, 4 °C), and supernatants reacted with chromogen (Elabscience Kit E-BC-K881-M). Absorbance was read at 520 nm (tissues) or 593 nm (cells).

### ROS detection

Cells incubated with 2 µM dihydroethidium (DHE) in serum-free medium (30 min, dark), washed, and imaged (excitation/emission: 488/610 nm).

### FerroOrange staining

Cells treated with 1 µM FerroOrange (30 min, dark), rinsed, and visualized (549/565 nm).

### Antioxidant and lipid peroxidation assays

#### GSH

Lysates reacted with DTNB-containing reagent (25 min, dark), absorbance measured at 412 nm against a 0–50 µM standard curve.

#### MDA

Samples incubated with thiobarbituric acid (15 min, 95 °C), cooled, and quantified at 532 nm using tetraethoxypropane standards.

#### SOD

Supernatants mixed with WST-8 reagent (30 min, dark), absorbance at 450 nm correlated with SOD activity (0–20 U/mL curve).

#### SOD activity assay

Supernatants reacted with WST-8 reagent and xanthine oxidase (30 min, 37 °C, dark), absorbance measured at 450 nm. SOD activity calculated based on inhibition percentage and normalized to protein concentration (U/mg protein) or tissue weight (U/g).

#### ATP content assay

Lysates mixed with Reaction Buffer containing firefly luciferase and luciferin, luminescence intensity (RLU) measured immediately. ATP concentrations determined against a 1 nM–10 µM standard curve and normalized to protein concentration (nmol/mg protein) or tissue weight (nmol/g).

#### JC-1

Stain cells with JC-1 dye, wash, then measure the red (J-aggregates, high membrane potential) versus green (monomers, low membrane potential) fluorescence by microscopy.

#### Ca^2+^

Load cells with the mitochondrial calcium indicator Rhod-2 AM, wash, and then measure red fluorescence by or fluorescence microscopy to assess mitochondrial Ca^2+^ levels.

### Transmission electron microscopy (TEM)

HT22 hippocampal neurons were chemically immobilized with 2% glutaraldehyde (4 °C, 24 h), subjected to graded ethanol dehydration, and polymerized in Spurr’s epoxy resin. Semithin sections (70 nm thickness) were analyzed by transmission electron microscopy (Hitachi H7800).

### Immunofluorescence

Sections or cells were permeabilized with 0.5% Triton X-100, then blocked with goat serum, and subsequently incubated overnight with either anti-GPX4 antibody (1:100, sc-166570) or anti-ACSL4 antibody (1:200, S-461-6). Secondary antibodies (1:500, SA00013-series) and DAPI counterstaining were applied prior to fluorescence imaging.

### Western blotting

Protein lysates (Beyotime BCA kit) were resolved on 10% SDS-PAGE, transferred to PVDF membranes, and probed with antibodies against GPX4 (1:1,000), ACSL4 (1:1,000), SLC7A11 (1:1,000), NCOA4 (1:1,000), and β-actin (1:5,000). HRP-conjugated secondaries and ECL were used for chemiluminescent detection (ImageJ quantification).

### Statistical analysis

Data expressed as mean ± SD. Parametric tests (Student’s *t*/ANOVA) applied after confirming normality (Shapiro-Wilk) and homoscedasticity (Levene). Non-parametric alternatives (Mann-Whitney/Kruskal–Wallis) used when assumptions violated. Correlation and interaction analyses performed with appropriate corrections. Analyses conducted using SPSS 27.0 and GraphPad Prism 9.5 (α = 0.05).

## Result

### *In Vivo* Experiments.

#### Chronic stress exacerbates behavioral alterations in diabetic rats

**FIGURE 1:**
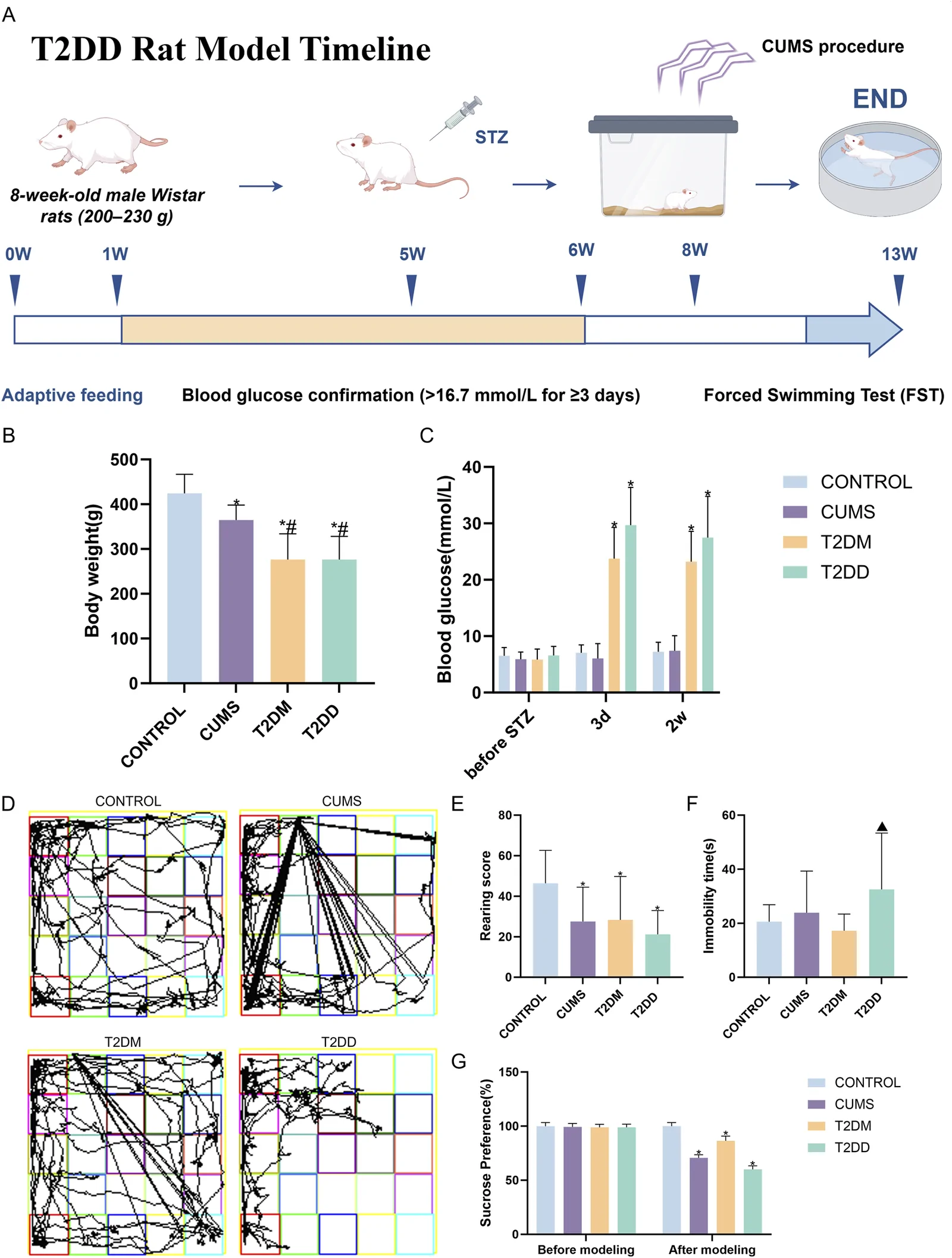
Chronic stress exacerbates behavioral alterations in diabetic rats. **(A)** Timeline diagram of the rat modeling process. **(B)** Body weight changes after model induction. **(C)** Fasting blood glucose levels at baseline, day 3, and week 2 post-STZ injection. **(D)** Representative open field test (OFT) trajectories. **(E)** Quantitative analysis of rearing frequency and central zone dwell time in OFT. **(F)** Immobility time in the forced swimming test (FST). **(G)** Sucrose preference in the sucrose preference test (SPT). Data are presented as mean ± SD. *P < 0.05 vs. control group; #P < 0.05 vs. CUMS group.

Timeline diagram of the rat modeling process are show in Figure 1A. Body weight changes post-model induction are shown in Figure 1B. Both the T2DM and T2DD groups showed significant weight loss compared with the control group (P < 0.05). The T2DD group had a further decrease in body weight relative to the CUMS group (P < 0.05), whereas no statistically significant difference was found between the T2DM and T2DD groups (Figure 1B). Blood glucose levels during model establishment are presented in Figure 1C. Baseline glucose levels were <16.7 mmol/L across all groups prior to STZ induction, with no intergroup differences. Following STZ administration, fasting blood glucose in T2DM and T2DD groups exceeded 16.7 mmol/L at all time points (Day 3 and Week 2; P < 0.05 vs. control), while CUMS and control groups remained comparable (Figure 1C). OFT trajectories are displayed in Figure 1D. Rats in CUMS, T2DM, and T2DD groups demonstrated reduced exploration of the central zone, with a preference for peripheral regions compared with controls (Figure 1D). Rearing frequency and central zone dwell time in the OFT are quantified in Figure 1E. T2DD rats exhibited fewer spontaneous rearings and prolonged central zone occupancy (P < 0.05). Immobility time in the FST is summarized in Figure 1F. CUMS, T2DM, and T2DD groups showed increased immobility compared with controls, though these differences lacked statistical significance. Conclusion: Chronic stress exacerbates behavioral deficits in diabetic rats. As shown in Figure 1G, sucrose preference was markedly reduced in the T2DD group (60%) compared with the CONTROL group (100%) after modeling, while CUMS and T2DM groups showed moderate reductions, indicating that the comorbid condition exacerbates depressive-like behavioral deficits. Successfully modeled rats were selected for subsequent experiments (Figures 1B–G).

#### Chronic stress exacerbates neuronal injury in the primary somatosensory cortex of diabetic rats

**FIGURE 2:**
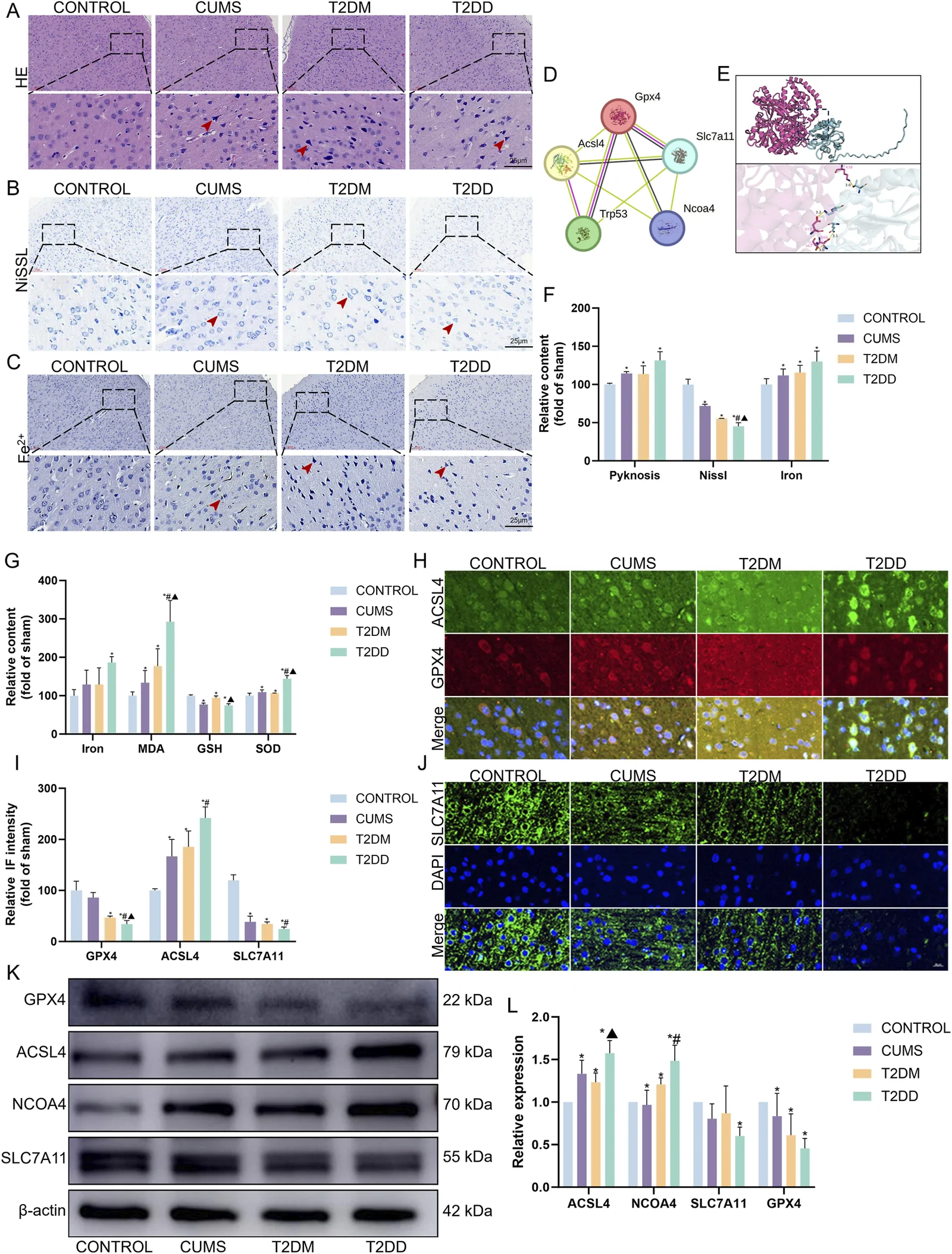
Chronic stress exacerbates neuronal injury and ferroptosis in the primary somatosensory cortex of diabetic rats. **(A)** Representative H&E staining images showing pyknotic neurons (arrows). **(B)** Representative Nissl staining images showing Nissl body loss. **(C)** Fe^2+^ deposition detected by Fe^2+^ staining (brown signals). **(D)** Protein-protein interaction (PPI) network of ferroptosis-related proteins (Gpx4, Slc7a11, Acsl4, Trp53, Ncoa4). **(E)** Structural modeling of the Gpx4-ACSL4 complex showing hydrogen bond interactions. **(F)** Quantitative analysis of pyknotic neurons, Nissl bodies, and Fe^2+^-positive neurons per high-power field (HPF). **(G)** Levels of Fe^2+^, MDA, GSH, and SOD in cortical tissues. **(H)** Immunofluorescence staining of ACSL4. **(I)** Quantitative analysis of ACSL4 fluorescence intensity and Quantitative analysis of SLC7A11 fluorescence intensity. **(J)** Immunofluorescence staining of SLC7A11. **(K)** Western blot analysis of GPX4, ACSL4, SLC7A11, and NCOA4. **(L)** Quantitative analysis of protein expression levels. Data are presented as mean ± SD. *P < 0.05 vs. control group; #P < 0.05 vs. T2DM group; &P < 0.05 vs. CUMS group.

Neuronal damage in the primary somatosensory cortex was evaluated via H&E and Nissl staining to assess the impact of chronic stress on hyperglycemia-induced injury. H&E staining results are shown in Figure 2A. The primary somatosensory cortex of the control group exhibited intact neuronal architecture, while scattered pyknotic neurons were observed in CUMS and T2DM groups. As shown in Figure 2A, T2DD rats exhibited an even greater increase in pyknotic neurons. The number of pyknotic neurons per high-power field (HPF) was significantly elevated in both the T2DM and CUMS groups compared with the control group (P < 0.05). Although T2DD rats showed a numerical increase in pyknotic neurons compared with T2DM and CUMS groups, this difference was not statistically significant. Nissl staining results are presented in Figure 2B. Control rats exhibited abundant, deeply stained Nissl bodies. In contrast, CUMS and T2DM groups showed lighter and fragmented Nissl body staining, while Figure 2B. Nissl bodies were nearly absent in T2DD rats (Figure 2B). Quantitatively, Nissl body counts were significantly reduced in CUMS and T2DM groups compared with controls (P < 0.05). Control neurons displayed normal morphology, whereas CUMS and T2DM groups exhibited diffuse granular Fe^2+^ deposits in the cytoplasm. Cortical Fe^2+^ deposition is illustrated in Figure 2C. T2DD rats showed reduced neuronal counts, increased Fe^2+^-positive neurons, and vacuolar degeneration (Figure 2C). T2DD rats demonstrated further reductions in Nissl body numbers relative to CUMS and T2DM groups (P < 0.05). The number of Fe^2+^-positive neurons per HPF was significantly elevated in T2DM, CUMS, and T2DD groups compared with controls (P < 0.05). Conclusion: Chronic stress exacerbates neuronal injury in the primary somatosensory cortex of diabetic rats, as evidenced by increased pyknotic neurons, Nissl body loss, and Fe^2+^ accumulation (Figure 2F).

Protein-protein interaction and structural analysis of key ferroptosis regulators are shown in Figures 2D,E.Protein-protein interaction (PPI) network of five core ferroptosis-related proteins: Gpx4, Slc7a11, Acsl4, Trp53, and Ncoa4. Nodes represent individual proteins, and edges indicate functional interactions supported by different evidence sources (color-coded) (Figure 2D). Structural modeling of the Gpx4–ACSL4 complex. Top: Overall binding interface of Gpx4 (pink) and ACSL4 (cyan). Bottom: Close-up view of the key interacting residues, showing hydrogen bonds and corresponding distances (Figure 2E).

### Chronic stress exacerbates oxidative stress and Cortical Ferroptosis in a diabetic rat model

Figure 2G presents the Fe^2+^ levels measured in the brain tissues of the rat models. Relative to the control group, the T2DD group showed a significant increase in Fe^2+^ content (P < 0.05); similarly, both the CUMS and T2DM groups also had elevated Fe^2+^ levels compared with the control group (P < 0.05) (Figure 2G).

We next examined oxidative stress markers, including MDA, GSH, and SOD. The statistical results for MDA, GSH, and SOD content are shown in Figure 2G. MDA levels were significantly higher in the T2DM and CUMS groups than in the control group (P < 0.05), and the T2DD group exhibited an even more pronounced increase compared with both the T2DM and CUMS groups (P < 0.05). GSH levels were significantly reduced in the CUMS, T2DM, and T2DD groups relative to the control group (P < 0.05), with the T2DD group showing a greater decrease than the T2DM group (P < 0.05). Notably, SOD levels were significantly elevated in the CUMS, T2DM, and T2DD groups compared with the control group (P < 0.05), and the T2DD group had a higher increase than both the T2DM and CUMS groups (P < 0.05).

Based on these findings, we investigated the expression levels of key ferroptosis-related proteins GPX4, ACSL4, NCOA4, and SLC7A11. Figure 2H shows the immunofluorescence staining of ACSL4, and Figure 2J displays the immunofluorescence staining of SLC7A11. Compared with the control group, the fluorescence intensity of ACSL4 was increased in the T2DM, CUMS, and T2DD groups, while the fluorescence intensity of SLC7A11 gradually decreased in these experimental groups (Figure 2I). Figure 2K presents the immunoblotting results of ferroptosis-related proteins, and Figure 2L show the quantitative analyses. Regarding GPX4, its relative expression level was significantly lower in the T2DM, CUMS, and T2DD groups than in the control group (P < 0.05). For ACSL4, the relative expression level was markedly higher in the CUMS, T2DM, and T2DD groups compared with controls (P < 0.05), with the T2DD group showing a further increase relative to the T2DM group (P < 0.05). As for SLC7A11, its relative expression was significantly reduced in the T2DD group versus the control group (P < 0.05), whereas no statistically significant difference was observed in the CUMS or T2DM groups. Concerning NCOA4, the relative expression level was significantly upregulated in the T2DM, CUMS, and T2DD groups compared with the control group (P < 0.05). The T2DD group had higher NCOA4 levels than the CUMS group (P < 0.05), but no significant difference was found between the T2DD and T2DM groups. These findings suggest that chronic stress may exacerbate neuronal damage in the cortical region of the T2DD group by intensifying ferroptosis.

### *In vitro* experiments

#### High glucose and corticosterone synergistically enhance oxidative stress and induce ferroptosis in HT22 cells

**FIGURE 3:**
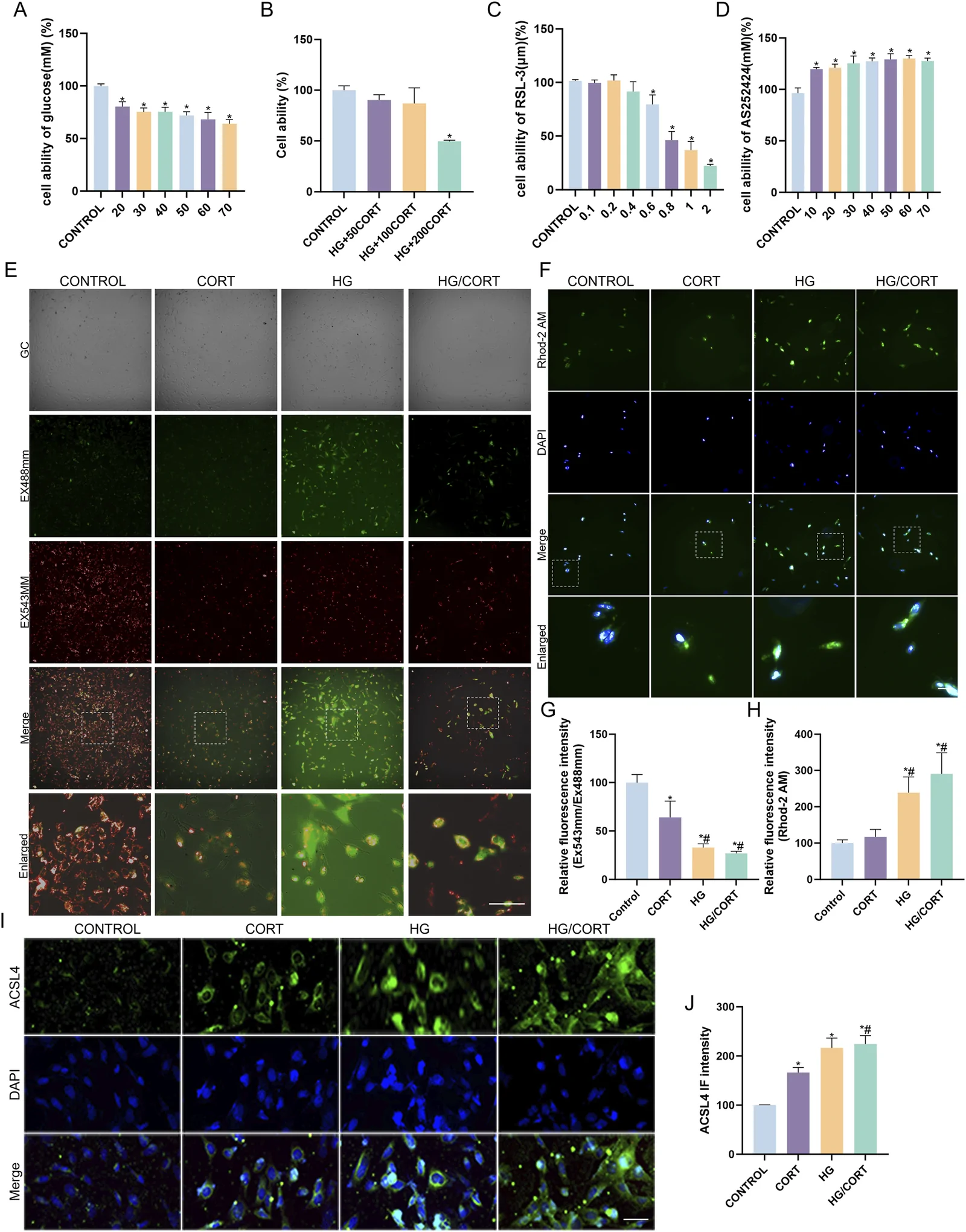
High glucose and corticosterone synergistically induce oxidative stress and ferroptosis in HT22 cells. **(A)** Cell viability under increasing glucose concentrations. **(B)** Cell viability under 50 mM glucose combined with different corticosterone concentrations. **(C)** Cell viability under increasing RSL3 concentrations. **(D)** Cell viability under increasing AS252424 concentrations. **(E)** Bright-field images showing cell morphology and JC-1 staining for mitochondrial membrane potential (ΔΨm). **(F)** Rhod-2 AM staining for intracellular Ca^2+^ levels. **(G)** Quantitative analysis of the red/green fluorescence intensity ratio of JC-1. **(H)** Quantitative analysis of Rhod-2 AM fluorescence intensity. **(I)** Immunofluorescence staining of ACSL4. **(J)** Quantitative analysis of ACSL4 fluorescence intensity. Data are presented as mean ± SD. *P < 0.05 vs. control group; #P < 0.05 vs. HG group; &P < 0.05 vs. CORT group.

To further validate whether chronic stress exacerbates ferroptosis in rats, we investigated the effects of combined high glucose (HG) and corticosterone (CORT) treatment on ferroptosis in mouse hippocampal HT22 cells through *in vitro* experiments. Figure 3A shows the statistical results of HT22 cell viability under different glucose concentrations. Compared with the control group, cell viability decreased significantly with increasing glucose concentrations (P < 0.05). We selected a glucose concentration that reduced cell viability to 50% for subsequent experiments (Figure 3A). Figure 3A illustrates the effects of 50 mM glucose combined with three different corticosterone concentrations. The results demonstrated that the 200 μM corticosterone treatment caused the most significant reduction in cell viability (P < 0.05) (Figure 3B). Figure 3C presents the statistical results of HT22 cell viability treated with the GPX4 inhibitor RSL3 at different concentrations, and Figure 3D shows the effects of the ACSL4 inhibitor AS252424. As shown in Figure 3C, cell viability decreased markedly with increasing RSL3 concentrations, reaching the lowest level at 0.8 μM (P < 0.05). Conversely, cell viability increased with higher AS252424 concentrations, peaking at 10 mM (P < 0.05) (Figure 3D).

To further investigate the mechanisms underlying mitochondrial dysfunction in HG/CORT-treated HT22 cells, we assessed mitochondrial membrane potential (ΔΨm) and intracellular Ca^2+^ levels using JC-1 and Rhod-2 AM staining, respectively. As shown in the bright-field images in Figure 3E, the Control group exhibited normal neuronal morphology with intact cell bodies and well-extended neurites. In contrast, the CORT and HG groups showed progressive cell shrinkage, reduced neurite outgrowth, and an increased number of floating dead cells. The HG/CORT group displayed the most severe morphological alterations, characterized by marked cell rounding, pronounced neurite retraction, and extensive detachment from the culture surface, indicating synergistic cytotoxic effects of high glucose and corticosterone on HT22 neurons. As shown in the JC-1 staining images in Figure 3E, the Control group exhibited predominantly red fluorescence (JC-1 aggregates), indicating intact ΔΨm. In contrast, the CORT and HG groups showed increased green fluorescence (JC-1 monomers) accompanied by reduced red fluorescence, suggesting mitochondrial depolarization. The HG/CORT group displayed the most pronounced shift from red to green fluorescence, with a marked decrease in the red/green fluorescence intensity ratio compared with the Control group (P < 0.05). Quantitative analysis further revealed that the HG/CORT group exhibited a significantly lower ratio than both the HG and CORT groups (P < 0.05). These findings indicate that high glucose and corticosterone synergistically disrupt mitochondrial membrane potential (Figure 3G).

We next examined intracellular Ca^2+^ levels using Rhod-2 AM staining. As presented in Figure 3F, Rhod-2 AM fluorescence intensity was weakly detected in the Control group, while CORT and HG treatments alone induced moderate increases in Ca^2+^ levels. Notably, combined HG/CORT treatment resulted in the most pronounced elevation of Rhod-2 AM fluorescence, and quantitative analysis confirmed that Ca^2+^ levels were significantly increased in the HG/CORT group compared with the Control, HG, and CORT groups (P < 0.05). These results suggest that hyperglycemia and chronic stress synergistically disrupt intracellular calcium homeostasis, which may contribute to mitochondrial dysfunction and ferroptosis in T2DD-associated neuronal injury (Figure 3H).

We assessed ferroptosis-related proteins in HG/CORT-treated HT22 cells using immunofluorescence and immunoblotting. Figure 3I shows the ACSL4 fluorescence intensity. Compared with the control group, the fluorescence intensity of ACSL4 increased in the CORT, HG, and HG/CORT groups. Figure 3J provide the quantitative analyses.

**FIGURE 4:**
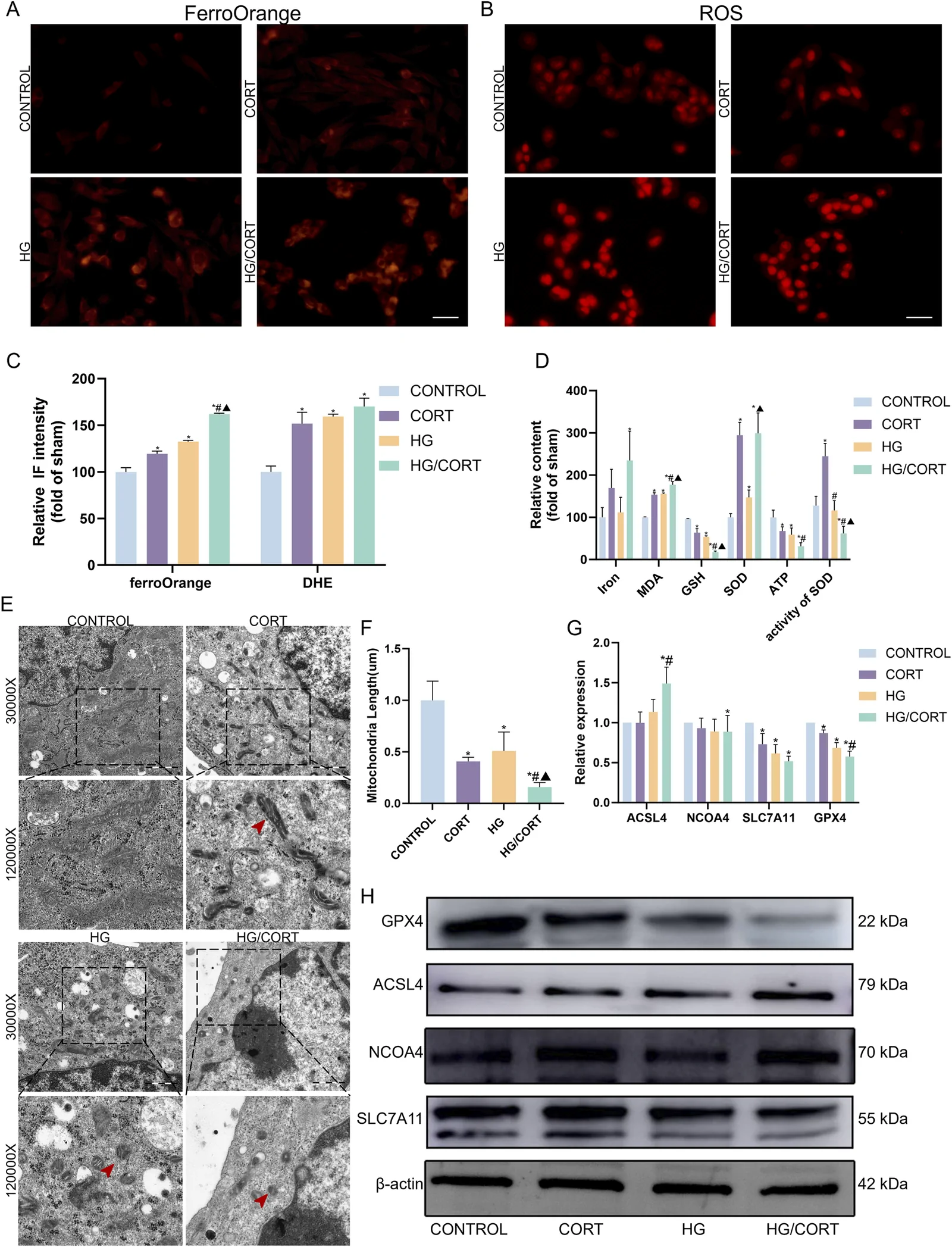
High glucose and corticosterone exacerbate mitochondrial damage and ferroptosis in HT22 cells. **(A)** Representative FerroOrange staining for Fe^2+^ detection. **(B)** Representative DHE staining for ROS detection. **(C)** Quantitative analysis of ROS and Fe^2+^ fluorescence intensity. **(D)** Levels of Fe^2+^, MDA, GSH, ATP, and SOD activity in HT22 cells. **(E)** Transmission electron microscopy (TEM) images of mitochondrial ultrastructure. **(F)** Quantification of mitochondrial length. **(G)** Quantitative analysis of protein expression levels. **(H)** Western blot analysis of GPX4, ACSL4, SLC7A11, and NCOA4. Data are presented as mean ± SD. *P < 0.05 vs. control group; #P < 0.05 vs. HG/CORT group; &P < 0.05 vs. RSL3 group.

After treating HT22 cells with high glucose (HG) and corticosterone (CORT), we measured intracellular ROS and Fe^2+^ levels using DHE and Fe^2+^ staining. ROS (reactive oxygen species) are oxygen-containing free radicals and peroxides associated with oxygen metabolism, and FerroOrange is a specific fluorescent probe for Fe^2+^. As shown in Figures 4A–C, which present the ROS and Fe^2+^ levels in HT22 cells, both the CORT and HG groups exhibited increased fluorescence intensities for ROS and Fe^2+^ compared with the control group. The HG/CORT group exhibited further elevated ROS and Fe^2+^ fluorescence intensities compared with the CORT and HG groups (Figure 4C).

#### High glucose and corticosterone synergistically aggravate oxidative stress and induce mitochondrial structural damage in HT22 cells

We further analyzed oxidative stress markers in HT22 cells.

Figure 4D presents the statistical results for Fe^2+^ content, as well as MDA, SOD, GSH, ATP, and SOD activity levels in HT22 cells. Compared with the control group, Fe^2+^ levels in the CORT and HG groups showed a rising trend, though this difference was not statistically significant. In contrast, the HG/CORT group exhibited a significant increase in Fe^2+^ content relative to the control group (P < 0.05). MDA levels were significantly elevated in the CORT and HG groups compared with controls (P < 0.05), with an even greater increase in the HG/CORT group than in either the CORT or HG group (P < 0.05). GSH levels were significantly reduced in the CORT, HG, and HG/CORT groups compared with the control group (P < 0.05), and the HG/CORT group showed a larger reduction than the CORT and HG groups (P < 0.05), suggesting that iron imbalance worsens oxidative stress in the HG/CORT group. Because a paradoxical rise in SOD levels was observed in the animal model, we also measured SOD content *in vitro*. Figure 4D shows that SOD levels were significantly increased in the CORT, HG, and HG/CORT groups relative to the control group (P < 0.05). The HG/CORT group had a further increase compared with the HG group (P < 0.05), but no statistically significant difference was found between the CORT and HG/CORT groups. ATP levels, reflecting mitochondrial energy production, were significantly reduced in the CORT and HG groups compared with the control group (P < 0.05), with the HG/CORT group exhibiting a more pronounced decrease than both the CORT and HG groups (P < 0.05), indicating that combined HG/CORT treatment synergistically impairs mitochondrial bioenergetic function. Regarding SOD activity, a similar paradoxical elevation was observed: SOD activity was significantly increased in the CORT group relative to the control group (P < 0.05), while the HG group showed no statistically significant difference compared with the control group. The HG/CORT group exhibited a further increase compared with the CORT group (P < 0.05). However, no statistically significant difference was found between the HG and HG/CORT groups. These findings suggest that the increased SOD content may represent a compensatory adaptive response to impaired enzyme function, which fails to counteract the concurrent GSH depletion and mitochondrial dysfunction in HG/CORT-treated cells.

Mitochondrial ultrastructure in HT22 cells was evaluated by TEM. Figure 4E displays TEM images of HT22 cell mitochondria. The control group showed normal mitochondrial morphology with intact plasma membranes and regularly arranged cristae. In contrast, the CORT and HG groups exhibited varying degrees of mitochondrial cristae fragmentation, dissolution, and vacuolation compared with controls. The HG/CORT group displayed more pronounced alterations in mitochondrial morphology compared with the CORT and HG groups (Figures 4E,F).

Using immunoblotting, we examined ferroptosis-related proteins in HG/CORT-treated HT22 cells (Figure 4H), with quantitative analyses shown in Figure 4G. As illustrated in Figure 4G, the relative expression of GPX4 was decreased in the CORT, HG, and HG/CORT groups compared with the control group (P < 0.05). The HG/CORT group exhibited a more pronounced reduction than the CORT group (P < 0.05), but no statistically significant difference was observed between the HG and HG/CORT groups. Figure 4G also shows that SLC7A11 expression was reduced in the CORT, HG, and HG/CORT groups relative to controls (P < 0.05). Although the HG/CORT group showed a further decrease compared with both the CORT and HG groups, this difference was not statistically significant. Co-treatment with HG and CORT significantly upregulated ACSL4 and NCOA4 expression. As presented in Figure 4G, ACSL4 expression was markedly increased in the CORT, HG, and HG/CORT groups versus the control group (P < 0.05). The HG/CORT group had higher ACSL4 levels than the CORT group (P < 0.05), while no significant difference was found between the CORT and HG/CORT groups. Regarding NCOA4, its relative expression was significantly elevated only in the HG/CORT group compared with the control group (P < 0.05), with no statistical differences noted in the CORT or HG groups alone.

#### ACSL4 inhibition attenuates RSL3-Induced oxidative stress and ferroptosis in HT22 cells

*In vitro* experiments, we explored the mechanisms using the GPX4-targeted inhibitor RSL3 and the ACSL4 inhibitor AS252424.

**FIGURE 5:**
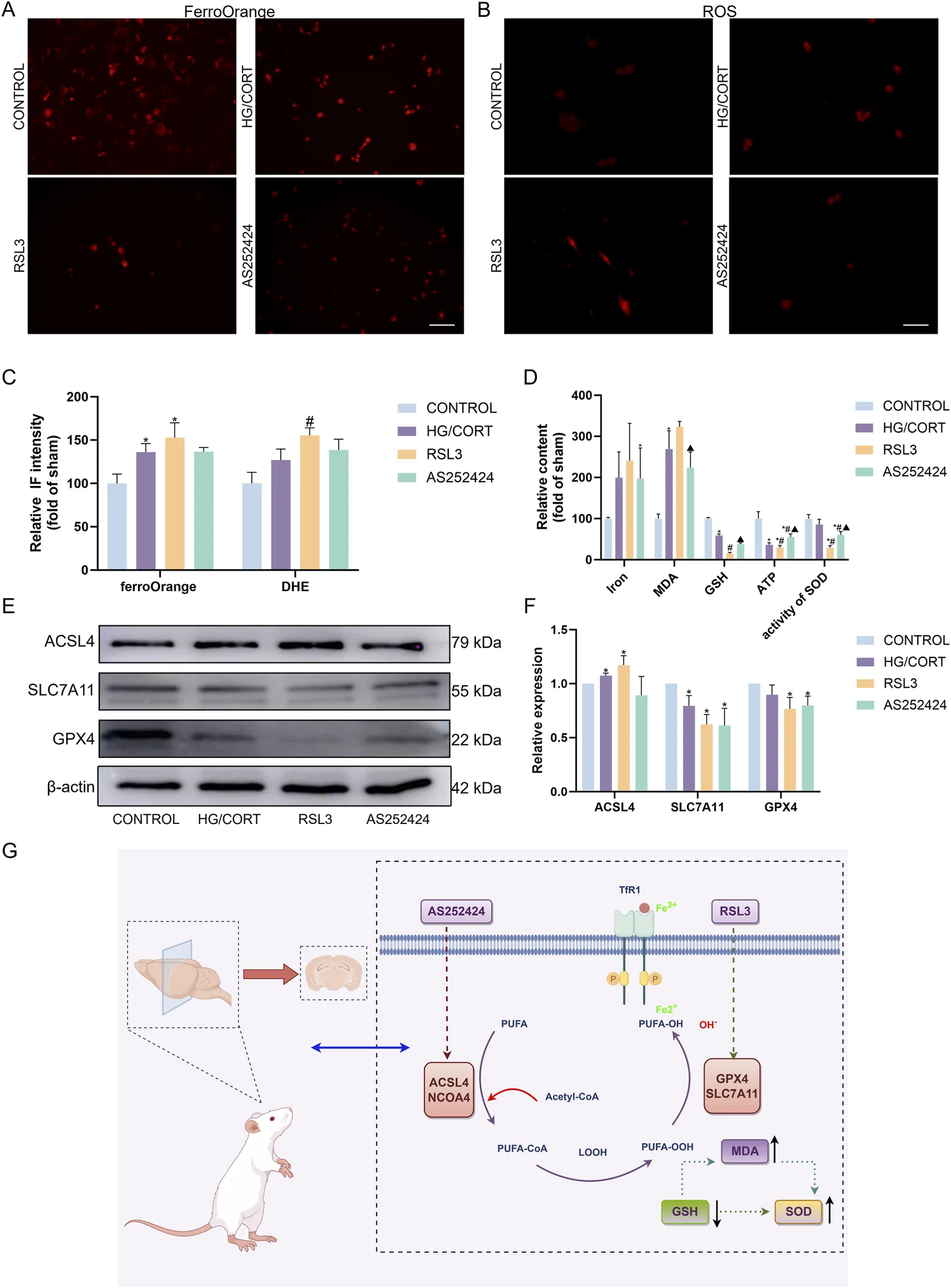
Inhibition of ACSL4 attenuates RSL3-induced ferroptosis in HT22 cells. **(A)** Representative FerroOrange staining for Fe^2+^ detection. **(B)** Representative DHE staining for ROS detection under RSL3 and AS252424 treatment. **(C)** Quantitative analysis of ROS and Fe^2+^ fluorescence intensity. **(D)** Levels of Fe^2+^, MDA, GSH, ATP, and SOD activity under different treatments. **(E)** Western blot analysis of GPX4, ACSL4, and SLC7A11. **(F)** Quantitative analysis of protein expression levels. **(G)** Mechanistic overview of the GPX4/ACSL4 axis in regulating ferroptosis in T2DD models. Data are presented as mean ± SD. *P < 0.05 vs. control group; #P < 0.05 vs. HG/CORT group; &P < 0.05 vs. RSL3 group.

Figures 5A–C displays the ROS and Fe^2+^ levels. Compared with the control group, the HG/CORT group exhibited increased ROS and Fe^2+^ fluorescence intensities, which were further elevated in the RSL3 group. The AS252424 treatment reversed this trend. Figure 5D shows the statistical results of Fe^2+^ content, MDA, GSH, ATP, and SOD activity levels. Compared with the control group, Fe^2+^ levels were significantly increased in the AS252424 group (P < 0.05), whereas no statistical differences were observed in the HG/CORT or RSL3 groups. MDA levels were significantly elevated in the HG/CORT group relative to the control group (P < 0.05), with a further upward trend in the RSL3 group (though not statistically significant). When compared with the RSL3 group, the AS252424 group exhibited a marked reduction in MDA content (P < 0.05). GSH levels were significantly decreased in the HG/CORT group versus the control group (P < 0.05), and the RSL3 group showed an even greater reduction compared with the HG/CORT group (P < 0.05). Treatment with AS252424 significantly increased GSH content relative to the RSL3 group (P < 0.05). ATP levels, reflecting mitochondrial energy production, were significantly reduced in the HG/CORT and RSL3 groups compared with the control group (P < 0.05), with the RSL3 group exhibiting a more pronounced decrease than the HG/CORT group (P < 0.05). Notably, AS252424 treatment significantly restored ATP levels relative to the RSL3 group (P < 0.05), suggesting that ACSL4 inhibition partially rescues mitochondrial bioenergetic function. Regarding SOD activity, a similar compensatory elevation pattern was observed: SOD activity was significantly decreased in the HG/CORT and RSL3 groups compared with the control group (P < 0.05). The RSL3 group exhibited the lowest SOD activity among all groups, while AS252424 treatment significantly restored SOD activity compared with the RSL3 group (P < 0.05), indicating that ACSL4 inhibition alleviates the oxidative stress response. These findings indicate that inhibition of ACSL4 alleviates RSL3-induced oxidative stress and mitochondrial dysfunction in HT22 cells.

We further evaluated the expression levels of GPX4, ACSL4, and SLC7A11 in RSL3-treated HT22 cells via immunoblotting. Figure 5E shows the immunoblotting results, and Figure 5F provide the quantitative analyses. As shown in Figure 5F, the HG/CORT group exhibited reduced GPX4 expression (not statistically significant) and significantly decreased SLC7A11 levels versus the control group (P < 0.05), while ACSL4 expression was markedly upregulated (P < 0.05). Relative to the RSL3 group, AS252424 treatment led to increased expression of GPX4 and SLC7A11 as well as decreased ACSL4 levels, although these differences were not statistically significant. RSL3 treatment reduced the expression of GPX4 and SLC7A11, and AS252424 partially reversed this reduction.

Mechanistic schematic illustrating the regulation of ferroptosis by the GPX4/ACSL4 axis in HT22 hippocampal neurons under diabetic and depressive conditions, and the differential effects of pharmacological inhibition of GPX4 (RSL3) versus ACSL4 (AS252424) on ferroptotic signaling (Figure 5G). In the untreated rat model, ferroptosis is activated through coordinated dysregulation of the GPX4/ACSL4 axis: ACSL4—cooperatively with NCOA4—catalyzes the esterification of polyunsaturated fatty acids (PUFAs) into PUFA-CoA, thereby promoting lipid peroxidation and subsequent malondialdehyde (MDA) accumulation; concurrently, the GPX4/SLC7A11/glutathione (GSH) antioxidant axis functions to detoxify lipid hydroperoxides. Phosphorylation of transferrin receptor 1 (TfR1) enhances cellular Fe^2+^ uptake, further potentiating iron-dependent oxidative damage. Pharmacological inhibition of ACSL4 with AS252424 suppresses PUFA-CoA biosynthesis by disrupting ACSL4/NCOA4 activity, thereby attenuating ferroptotic cell death. In contrast, GPX4 inhibition by RSL3 impairs the GPX4/SLC7A11/GSH axis, depletes intracellular GSH, and exacerbates lipid peroxidation. Notably, a paradoxical elevation in SOD activity was consistently observed across all experimental conditions, llikely representing a compensatory response to severe oxidative stress rather than an effective antioxidant defense, as it failed to counteract the concurrent GSH depletion and GPX4 inactivation.

## Discussion

T2DD is a mental health disorder occurring in diabetic patients, characterized by the comorbidity of diabetes and depression. Hyperglycemic patients are more prone to developing depression. Studies suggest that diabetes induces central neuronal damage through pathways such as autophagy, inflammation, vagal nerve modulation, and ferroptosis, while depression exacerbates these pathological changes (Xu et al., 2023; Liu et al., 2023).

Current research on neuronal injury and behavioral alterations primarily focuses on hippocampal subregions and the prefrontal cortex. Wang et al. found that hippocampal neuronal ferroptosis contributes to cognitive dysfunction in a T2DM model. Zhang et al. observed varying degrees of neuronal atrophy in the prefrontal cortex of type 2 diabetic patients (Zhang et al., 2019). Chronic stress induces dendritic atrophy and spine loss in hippocampal and prefrontal cortical neurons (Qiao et al., 2016; Yi et al., 2012). In morphine-induced rat addiction models, alterations in cortical glucose metabolism and regional blood flow were observed (Shao et al., 2023). Additionally, reducing tremor-induced activation in the primary motor cortex enhanced the therapeutic efficacy of dopaminergic drugs (Nelson et al., 2018). These findings suggest that cortical changes may influence behavioral outcomes. In our study, the T2DD group exhibited more severe depressive-like behaviors, increased pyknotic neurons, and reduced Nissl bodies compared with the T2DM or CUMS groups. *In vitro* experiments demonstrated that combined HG/CORT treatment further reduced HT22 cell viability and exacerbated mitochondrial structural damage (e.g., cristae fragmentation and membrane disruption), indicating that mitochondrial dysfunction may be a key pathological basis for neuronal injury. These results suggest that depressive-like behaviors are closely associated with neuronal damage in the primary somatosensory cortex (Feng et al., 2024; Yao et al., 2020).

Ferroptosis, a regulated cell demise pathway mechanistically divergent from classical apoptosis, accidental necrosis, and autophagic processes, is fundamentally mediated through iron-catalyzed phospholipid peroxidation cascades (Pu et al., 2022). Both diabetes and depression are associated with reduced GPX4 expression and iron metabolism imbalance. However, the pathogenic mechanisms linking iron dyshomeostasis to T2DD remain unclear.

Chronic stress combined with hyperglycemia induces intracellular Fe^2+^ overload and excessive ROS generation. Our animal and cellular models revealed significant alterations in ferroptosis markers. The increased ROS initiate lipid peroxidation and markedly elevated MDA levels, and MDA is an important biomarker of oxidative damage and ferroptosis in neural cells. In the T2DD and HG/CORT groups, excessive iron accumulation, decreased GPX4 and SLC7A11 expression, and weakened glutathione-dependent lipid peroxidation clearance were observed. As the core endogenous antioxidant, GSH synthesis is suppressed due to the downregulation of SLC7A11, leading to GPX4 inactivation and further accumulation of lipid hydroperoxides. Iron overload upregulated ACSL4 and NCOA4, promoting polyunsaturated fatty acid esterification and increasing lipid peroxides and free iron. Elevated MDA and reduced GSH directly confirmed that lipid peroxidation drives ferroptosis in T2DD neurons.

Meanwhile, the elevated SOD level represents only a compensatory adaptive response to oxidative stress and cannot reverse GSH depletion and GPX4 dysfunction. The paradoxical elevation in SOD activity observed in the T2DD and HG/CORT groups likely represents a reduced enzyme activity response to overwhelming oxidative stress rather than a successful restoration of redox homeostasis. Similar paradoxical SOD elevations have been reported in early-stage diabetic neuropathy, neurodegenerative conditions, and chronic stress models, where the antioxidant system transiently upregulates superoxide scavenging in an attempt to counteract rising ROS levels. However, this adaptive response is insufficient to reverse GSH depletion or GPX4 inactivation, and may even exacerbate oxidative damage through excessive H_2_O_2_ production, ultimately failing to halt ferroptotic progression. (Chen et al., 2008; Zhao et al., 2024). This transient adaptive response does not necessarily indicate improved redox homeostasis, especially when coupled with reduced GPX4 activity and GSH depletion, which impair the clearance of lipid peroxides and perpetuate ferroptotic cascades.

The imbalance of elevated MDA, GSH and ATP depletion, and compensatory SOD upregulation collectively accelerates ferroptosis in sensory cortical neurons through dysregulation of the GPX4/ACSL4 axis.

GPX4 and ACSL4 antagonistically regulate lipid oxidation: GPX4 utilizes GSH to detoxify lipid peroxides, whereas ACSL4 esterifies polyunsaturated fatty acids into oxidation-prone phospholipids, inducing membrane damage. In T2DD models, hyperglycemia and chronic stress synergistically inhibit GPX4 and activate ACSL4, establishing a “lipid metabolism-ferroptosis” vicious cycle. Downregulated GPX4 impairs neuronal detoxification of lipid hydroperoxides (LOOH) (Wang Y. et al., 2024), while ACSL4 activation promotes pro-oxidative lipid conversion, amplifying oxidative damage and triggering ferroptosis. This process aggravates neuronal injury in the primary somatosensory cortex and may mediate synaptic inhibition, leading to anhedonia and cognitive deficits (Tan et al., 2024). The imbalance between GPX4 and ACSL4 creates a “oxidative damage-metabolic dysregulation” positive feedback loop, consistent with classical ferroptosis theory. Targeting this axis may restore redox homeostasis, offering novel therapeutic strategies.

In cellular experiments, treatment with the ACSL4 inhibitor AS252424 and GPX4 inhibitor RSL3 yielded critical insights: RSL3 suppressed GPX4 expression, while AS252424 partially reversed this effect. This indicates that ACSL4 inhibition restores GPX4 antioxidant capacity, reduces lipid peroxidation, improves iron homeostasis, and enhances antioxidant defenses. Notably, RSL3 treatment also led to a reduction in GPX4 protein levels, which may reflect decreased protein stability or enhanced degradation secondary to the loss of its enzymatic activity, a phenomenon that warrants further investigation. These findings confirm the functional antagonism between GPX4 and ACSL4 in T2DD models—ACSL4 activation exacerbates oxidative damage via lipid metabolism, whereas ACSL4 inhibition rebalances redox homeostasis to counteract ferroptosis.

## Study limitations

This study has several limitations. First, we only examined neuronal histopathology in the primary somatosensory cortex, leaving other brain regions unassessed. Second, we measured only partial ferroptosis-related proteins in animal and HT22 cell models. Third, the use of RSL3 and AS252424 in HT22 cells lacks validation in genetic models (e.g., GPX4 conditional knockout mice or ACSL4-overexpressing cells). The PPI and structural modeling in this study serve only as bioinformatic support and cannot replace functional experimental validation; Thus, the mechanistic interplay between GPX4 and ACSL4 requires further exploration. Additionally, the paradoxical increase in SOD activity despite severe oxidative damage requires further exploration. Whether this reflects a failed compensatory mechanism, and whether prolonged SOD overactivation leads to secondary issues such as H_2_O_2_ accumulation or metal ion dyshomeostasis, remains to be investigated.

## Conclusion

In the successfully established rat model, the T2DD group exhibited significantly reduced spontaneous activity in the open field test, prolonged immobility time in the forced swim test, increased pyknotic neurons, elevated Fe^2+^ levels, enhanced oxidative stress, and aggravated ferroptosis in the cortical region compared with the CUMS and T2DM groups. *In vitro* experiments demonstrated that AS252424 alleviated RSL3-induced suppression of GPX4 expression. Our *in vivo* data reveal an association between GPX4/ACSL4 axis imbalance and aggravated ferroptosis in the T2DD rat model, while *in vitro* experiments demonstrate that pharmacological inhibition of ACSL4 alleviates RSL3-induced ferroptotic changes. These findings suggest that the GPX4/ACSL4 axis may mechanistically contribute to ferroptosis in T2DD, though further genetic validation in animal models is warranted. Our findings partially delineate the GPX4/ACSL4-mediated pathomechanisms contributing to cognitive dysfunction in T2DD, revealing that targeted modulation of this axis attenuates - but does not fully abolish - ferroptosis-associated oxidative injury. This partial mechanistic elucidation provides preliminary evidence for developing subtype-specific therapeutic regimens in T2DD management.

## Data Availability

The raw data supporting the conclusions of this article will be made available by the authors, without undue reservation.
